# Editorial: Translational research of occupational therapy and neurorehabilitation

**DOI:** 10.3389/fnhum.2024.1432073

**Published:** 2024-06-07

**Authors:** Ryouhei Ishii, Scott Alan Smith, Ryoichiro Iwanaga, Jing Xiang, Leonides Canuet, Hideki Miyaguchi, Hiroyuki Inadomi

**Affiliations:** ^1^Department of Occupational Therapy, Graduate School of Rehabilitation Science, Osaka Metropolitan University, Osaka, Japan; ^2^Department of Psychiatry, Osaka University Graduate School of Medicine, Osaka, Japan; ^3^Department of Applied Clinical Research, School of Health Professions, University of Texas Southwestern Medical Center, Dallas, TX, United States; ^4^Graduate School of Biomedical Sciences, Nagasaki University, Nagasaki, Japan; ^5^MEG Center, Division of Neurology, Cincinnati Children's Hospital Medical Center, Cincinnati, OH, United States; ^6^University Hospital Nuestra Senora del Rosario and Sanitas Zarzuela Hospital, Madrid, Spain; ^7^Department of Human Behavior Science of Occupational Therapy, Health Sciences Major, Graduate School of Biomedical and Health Sciences, Hiroshima University, Hiroshima, Japan; ^8^Occupational Therapy Major, University of Kochi Health Sciences, Kochi, Japan; ^9^Department of Advanced Occupational Therapy, Human Health Sciences, Graduate School of Medicine, Kyoto University, Kyoto, Japan

**Keywords:** translational research, occupational therapy, neurorehabilitation, electroencephalography (EEG), virtual reality (VR), aid for decision-making in occupation choice (ADOC), frontal midline theta rhythm (Fmθ), kinesthetic illusion induced by visual stimulation (KINVIS)

The American Occupational Therapy Association (AOTA) defines Occupational Therapy (OT) as “a health and rehabilitation profession that assists individuals of all ages who have an injury, illness, cognitive impairment, mental illness, developmental, learning, or physical disability to maximize their independence. OT is aimed at maximizing autonomy in all aspects of daily activities, assisting the person in all types of disabilities and giving meaning by means of organized and deliberate activity, known as 'occupation'. OT therapy sessions are focused on engaging patients with meaningful activities to achieve goals and achieve adequate levels of satisfaction, productivity and independence. Patients can thus experience increased competence, self-efficacy, independence, purpose and, above all, a greater sense of wholeness.

New research and emerging technologies are providing therapeutic strategies and new devices to the field of neurorehabilitation (NR) research and OT clinical practice. This Research Topic highlights some of the innovative research findings that these new technologies are bringing to OT and NR. The first section introduces new technological approaches in OT and NR for cognitive and psychiatric disorders. Next, the application of new technologies to the rehabilitation of motor function through motor imagery and neurofeedback is presented. Finally, studies of neuromodulation to assess and directly work with motor function are reported. All suggest that new technologies such as virtual reality (VR), non-invasive brain stimulation and robot-assisted training can increase the effectiveness of OT and NR strategies. Brain imaging techniques such as electroencephalography (EEG), event-related potentials (ERP), near-infrared spectroscopy (NIRS) and functional magnetic resonance imaging (fMRI) also provide non-invasive and objective outcome measures for OT and NR.

Tomori et al. (2012) introduced Aid for Decision-making in Occupation Choice (ADOC), a digital tool facilitating shared decision-making in occupational target planning, enhancing collaboration between therapists and patients. Tokuda et al. investigated the impact of self-choice of interesting occupations on cognitive processing and job-related satisfaction using ERP and ADOC, revealing a significant increase in P300 amplitude during self-selection of an interesting job, suggesting the importance of patient involvement in goal setting. Kubo et al. explored how prior knowledge of traffic signals influences behavioral responses and neural mechanisms using RT and ERP, uncovering Stroop-like interference between prior knowledge and actual signal meaning. Shiraiwa et al. studied the physiological mechanisms underlying the therapeutic effects of craft activities in occupational therapy, revealing increased parasympathetic and sympathetic activity during craft tasks, suggesting a correlation between cardiac autonomic function and frontal midline theta rhythm (Fmθ) activity. Tabira et al. investigated the relationship between attentional bias and psychological ratings in chronic low back pain (LBP) patients, highlighting associations between psychological factors, attentional bias, and pain-related outcomes, indicating the potential benefits of attentional bias modification training in LBP management.

Niki et al. conducted a pilot study investigating the efficacy of immersive virtual reality (iVR) reminiscence in alleviating anxiety in the elderly with cognitive decline. Their findings suggest that iVR reminiscence, particularly when using live-action pictures, may effectively reduce anxiety without causing serious side effects. Yamada and Sumiyoshi conducted a review on transcranial direct current stimulation (tDCS) and its potential in treating various psychiatric disorders. They discussed the neurobiological mechanisms underlying tDCS and its ability to modulate cortical excitability, neurotransmitter activity, and information processing efficiency in the brain. Koyanagi et al. investigated the utility of functional near-infrared spectroscopy (fNIRS) in diagnosing post-stroke depression (PSD). They found that depressed patients exhibited lower oxy-Hb integral values and reduced frontal lobe activation compared to non-depressed patients, suggesting fNIRS oxy-Hb as a useful diagnostic tool for PSD.

Moriuchi et al. explored the relationship between motor imagery (MI) ratings and neurophysiological ratings during combined MI and action observation (MI+AO) tasks. They found a significant positive correlation between MI quality and transcranial magnetic stimulation (TMS)-evoked motor evoked potentials (MEPs), suggesting that MI quality may reflect corticospinal tract excitability during MI+AO tasks. Iso et al. investigated the relationship between oxy-Hb concentration during a motor imagery (MI) task and the level of motor learning. They observed significant changes in oxy-Hb concentration in the supplementary motor area (SMA), suggesting that hemodynamic brain activity during MI tasks may correlate with the level of motor learning.

In their study, Aoyama et al. explored the efficacy of Kinesthetic Illusion induced by Visual Stimulation (KINVIS) as a standalone intervention for finger flexor spasticity in stroke patients. By conducting a single-session KINVIS intervention on 14 stroke patients, they observed significant improvements in Modified Ashworth Scale scores and active range of finger extension movements. Additional experimentation suggested potential neurophysiological changes underlying the reduction in spasticity, indicating the clinical significance of KINVIS in improving upper limb function post-stroke. Irie et al. investigated the neuroscientific basis of motor imagery (MI) intervention for children with developmental coordination disorder (DCD). Their review highlighted neural alterations associated with DCD, including decreased activity in the mirror neuron system and sensory integration regions. Additionally, they proposed MI methods involving action observation and visual-motor tasks to activate relevant brain regions, offering insights into effective intervention strategies for children with DCD.

Matsuda et al. examined whether the efficacy of mental practice (motor imagery training) could be enhanced by providing neurofeedback based on transcranial magnetic stimulation (TMS)-induced motor evoked potentials (MEP). Their study suggested that TMS-induced MEP-based neurofeedback might enhance the effect of mental practice, indicating a potential avenue for improving motor learning outcomes through combined interventions. Zhang and Fong investigated the modulatory effect of priming intermittent theta burst stimulation (iTBS) with continuous theta burst stimulation (cTBS) on sensorimotor oscillatory activities in healthy adults. Their findings suggested that priming iTBS with cTBS yielded similar effects to iTBS alone in enhancing sensorimotor event-related desynchronization induced by mirror visual feedback. However, priming iTBS demonstrated more pronounced enhancements in movement-related desynchronization, particularly in the high mu band, suggesting a potential advantage of this combined stimulation protocol for motor learning paradigms. Fong et al. investigated the immediate effects of mirror visual feedback (MVF) on motor execution in stroke patients and healthy individuals using EEG. They found significant suppression of high beta event-related desynchronization (ERD) over the contralateral motor area during unimanual arm movements with MVF, suggesting a shift in sensorimotor ERD toward the contralateral hemisphere induced by MVF.

Sagari et al. examined the effects of antagonistic tasks on prefrontal cortical cerebral hemodynamics in healthy adults. They found that complex antagonistic tasks led to a greater increase in prefrontal cortex oxygenated hemoglobin concentration compared to non-antagonistic tasks, highlighting the impact of task complexity on cerebral blood flow dynamics. Matsumoto A. et al. investigated the contribution of corticospinal excitability to anticipatory postural adjustments (APAs) in lower limb muscles during ballistic upper limb movements. They observed increased corticospinal excitability in the tibialis anterior muscle preceding the dart throwing movement, suggesting a role of the corticospinal pathway in APAs. Matsumoto T. et al. also explored the influence of goal-directed movement on ipsilateral primary motor cortex (ipsi-M1) excitability during unilateral finger tapping tasks. They found reduced short-interval intracortical inhibition (SICI) in the ipsi-M1 during visually guided finger movements, suggesting modulation of ipsi-M1 excitability during goal-directed tasks. Matsugi et al. investigated arterial pressure and heart rate responses to noisy galvanic vestibular stimulation (nGVS) during static supine and whole-body tilt in healthy older adults. They found that nGVS did not significantly affect arterial pressure or heart rate, indicating the safety of nGVS application in this population.

Johnson et al. emphasized the importance of considering memory consolidation and reconsolidation in rehabilitation, highlighting the need for further research to explore their role in enhancing learning and memory between rehabilitation sessions. They suggested that understanding these processes could lead to the development of more effective and long-lasting rehabilitation interventions. Translational research in occupational therapy and neurorehabilitation holds immense significance in enhancing the quality of life for individuals facing various cognitive, physical, and psychiatric challenges. By bridging the gap between cutting-edge research findings and practical clinical applications, translational research empowers therapists to adopt innovative strategies and technologies, thereby maximizing the effectiveness of therapeutic interventions.

Through the integration of emerging technologies such as virtual reality, noninvasive brain stimulation, and robot-assisted training, occupational therapists can revolutionize neurorehabilitation approaches. These technologies offer promising avenues for enhancing cognitive function, motor skills, and emotional wellbeing in patients with neurological conditions. Moreover, the development of digital tools like Aid for Decision-making in Occupation Choice (ADOC) facilitates shared decision-making between therapists and patients, fostering a collaborative approach to goal setting and rehabilitation planning. By leveraging tools like ADOC, therapists can personalize interventions to align with each patient's unique preferences and priorities, ultimately promoting greater satisfaction and engagement in therapy sessions.

Furthermore, neurophysiological studies exploring phenomena such as attentional bias modification and motor imagery shed light on the underlying mechanisms of therapeutic interventions. By elucidating the neural correlates of cognitive processes and behavioral responses, researchers can refine existing interventions and develop targeted strategies to address specific impairments more effectively. Additionally, the utilization of advanced imaging techniques like functional near-infrared spectroscopy (fNIRS) offers valuable insights into the neurological changes associated with conditions such as post-stroke depression. By employing fNIRS as a diagnostic tool, therapists can accurately assess cerebral function and tailor interventions to address mood disturbances, thereby facilitating more comprehensive rehabilitation outcomes.

Overall, translational research in occupational therapy and neurorehabilitation holds tremendous promise for advancing clinical practice and improving the lives of individuals with neurological disorders. By embracing innovation, collaboration, and evidence-based practice, therapists can continue to enhance the efficacy and accessibility of rehabilitation services, ultimately empowering patients to achieve greater independence, functionality, and quality of life.

## Author contributions

RIs: Conceptualization, Data curation, Formal analysis, Funding acquisition, Investigation, Methodology, Project administration, Resources, Software, Supervision, Validation, Visualization, Writing – original draft, Writing – review & editing. SS: Methodology, Writing – review & editing. RIw: Funding acquisition, Writing – original draft, Supervision. JX: Validation, Writing – review & editing, Conceptualization. LC: Software, Writing – review & editing, Funding acquisition. HM: Validation, Visualization, Writing – original draft, Investigation. HI: Investigation, Writing – original draft, Data curation.
